# Noninvasive prediction of the clinical benefit of immunotherapy in hepatocellular carcinoma

**DOI:** 10.1007/s00535-025-02251-x

**Published:** 2025-05-30

**Authors:** Atsushi Ono, C. Nelson Hayes, Ryoichi Miura, Tomokazu Kawaoka, Masataka Tsuge, Shiro Oka

**Affiliations:** 1 Department of Gastroenterology, Graduate School of Biomedical and Health Sciences, Hiroshima University, Hiroshima, 734-8551 Japan; 2 Liver Center, Hiroshima University Hospital, Hiroshima, Japan

**Keywords:** Hepatocellular carcinoma, Immune checkpoint inhibitor, Immune microenvironment, Biomarker, Prediction

## Abstract

Long-term survival following a diagnosis of hepatocellular carcinoma (HCC) is greatly diminished when transplantation and surgical resection are ruled out. Fortunately, the advent of immune checkpoint inhibitors (ICIs) has revolutionized the treatment of advanced unresectable HCC (uHCC), prolonging median survival by over a year. T lymphocytes normally eliminate neoplastic cells, but some tumors suppress this response by binding to immune checkpoint receptors. Blocking this interaction via ICIs restores immune-mediated targeting of cancer cells. While ICI-based combination immunotherapy is currently recommended as the first-line systemic therapy for uHCC, the objective radiological response rate remains limited to 20–30%, as not all tumors exploit this mechanism. Consequently, strategies are being explored to modulate the immune microenvironment into a "hot" environment more responsive to ICIs by combining local therapies such as transarterial chemoembolization, ablation, and radiation therapy. Therapeutic options have also expanded beyond ICIs, emphasizing the importance of selecting the most appropriate treatment. Therefore, the development of biomarkers capable of predicting the efficacy of immunotherapy is a priority. Direct evaluation of immune cell infiltration through biopsy is currently the most effective method but involves issues such as invasiveness and susceptibility to sampling bias. In this review, we aim to highlight promising non-invasive biomarkers and scoring systems that have the potential to improve treatment outcomes, including blood-based biomarkers such as lymphocyte ratios, cytokines, C-reactive protein, and alpha-fetoprotein; imaging biomarkers such as MRI, ultrasound, and contrast-enhanced CT; and other clinical indicators such as sarcopenia, grip strength, and diversity of the gut microbiome.

## Introduction

The advent of immune checkpoint inhibitors has changed the treatment of advanced unresectable hepatocellular carcinoma (uHCC). Current guidelines from American Society of Clinical Oncology (ASCO) [1], European Society for Medical Oncology (ESMO) [2], American Association for the Study of Liver Diseases (AASLD) [3] and Japan Society of Hepatology (JSH) [4] recommend first-line combination therapy with atezolizumab (anti-programmed death ligand 1 [PD-L1] antibody) + bevacizumab (anti-vascular endothelial growth factor [VEGF]-A antibody) (Atezo + Bev) or durvalumab (anti-PD-L1 antibody) + tremelimumab (anti-cytotoxic T lymphocyte–associated antigen 4 [CTLA4] antibody) (Durva + Treme) for uHCC patients. However, the fact that many patients do not respond effectively to these therapies is evident in clinical trials such as IMbrave150 [5], HIMALAYA [6], and CheckMate-9DW [7]. In these trials, the objective radiological response was confirmed in only 27.3%, 20.1%, and 36% of participants, respectively.

Against this background, efforts to enhance the response to immunotherapy by combining local therapies such as transarterial chemoembolization (TACE), ablation, and radiation therapy (RT) are currently being actively tested.

In addition, there are reports of the potential of lenvatinib, one of the systemic therapy options for advanced HCC, to modulate the immune microenvironment to be more favorable for immunotherapy [8, 9].

As such, predicting the efficacy of combined immunotherapy before treatment initiation is expected to become increasingly important in planning treatment strategies.

At present, the most effective methods for predicting treatment efficacy involve using biopsy tissues to evaluate immune cell infiltration within the tumor or extracting RNA from the tissue to perform transcriptome-based subclassification or gene expression signature analysis. For example, in the IMbrave150 study, cases with immune cell infiltration showed significant improvements in overall survival (OS) and progression-free survival (PFS) with Atezo + Bev compared to sorafenib, demonstrating treatment benefits. However, for so-called "cold" tumors, no significant prognostic improvement was observed with Atezo + Bev compared to the sorafenib group [10].

However, using biopsy tissues raises issues such as intratumoral and intertumoral heterogeneity. Moreover, as cancer continues to evolve, real-time evaluation at the time of switching therapies would be ideal, but repeated tumor biopsies are neither feasible due to invasiveness nor practical from a healthcare economics perspective.

Therefore, this review focuses on non-invasive biomarkers and aims to summarize the findings reported to date.

## Blood-based biomarkers

Candidates for blood-based biomarkers are summarized in Table 1.

**Table 1:** Blood based biomarkers

	Markers	Unfavorable	Treatment	*n*	Study design	PMID
Conventional parameters	AFP	≥ 100 ng/mL	Atezo + Bev	719	Retrospectively	PMID: 38577725
		≥ 100 ng/mL	Atezo + Bev	297	Retrospectively	PMID: 35749019
		≥ 100 ng/mL	Anti PD-(L)1-based immunotherapy	292	Retrospectively	PMID: 34648895
		≥ 100 ng/mL	Atezo + Bev	426	Retrospectively	PMID: 36076009
		High	ICI	5322	Meta-analysis	PMID: 36933492
	DCP	≥ 100 mAU/mL	Atezo + Bev	719	Retrospectively	PMID: 38577725
	CRP	≥ 1.0 mg/dL	Atezo + Bev	297	Retrospectively	PMID: 35749019
		≥ 1.0 mg/dL	anti PD-(L)1-based immunotherapy	292	Retrospectively	PMID: 34648895
	CRAFITY score	High	Atezo + Bev	302	Retrospectively	PMID: 39289234
		High	Atezo + Bev	297	Retrospectively	PMID: 35749019
		High	anti PD-(L)1-based immunotherapy	292	Retrospectively	PMID: 34648895
	alpha-FAtE	Low	Atezo + Bev	543	Retrospectively	PMID: 37994647
	IMABALI-De score	High	Atezo + Bev	719	Retrospectively	PMID: 38577725
	Modified albumin–bilirubin grade and α-fetoprotein (mALF) score	High	Atezo + Bev	426	Retrospectively	PMID: 36076009
	ALBI score	high	Ipilimumab combined with nivolumab or pembrolizumab	25	Retrospectively	PMID: 33563773
	ALBI grade	> 2 or ≥ 3	ICIs	341	Prospectively	PMID: 32664319
	mALBI grade	> 2a or ≥ 2b	Atezo + Bev	719	Retrospectively	PMID: 38577725
		2b or 3	Atezo + Bev	242	Retrospectively	PMID: 36070216
		2b or 3	Atezo + Bev	426	Retrospectively	PMID: 36076009
Immune-related biomarkers	Neutrophil-to-lymphocyte ratio (NLR)	≥ 5	Atezo + Bev	296	Retrospectively	PMID: 36497316
		≥ 3.20	Atezo + Bev	100	Retrospectively	PMID: 36883351
		> 3.22	Durva + Treme	228	Retrospectively	PMID: 36477555
		High	Durva + Treme	2406	Meta-analysis	PMID: 37300457
		High	ICI	5322	Meta-analysis	PMID: 36933492
		> 2.77	ICI	249	Retrospectively	PMID: 37322411
		> 5	ICI	362	Retrospectively	PMID: 35008350
		> 3	Atezo + Bev	885	Retrospectively	PMID: 39296620
		≥ 2.56	Atezo + Bev	242	Retrospectively	PMID: 36070216
	Platelets to lymphocytes ratio (PLR)	≥ 300	ICI	362	Retrospectively	PMID: 35008350
	Circulating immune index (CII)	> 43.1	ICI + lenvatinib	194	Retrospectively	PMID: 36910622
	IL-6	≥ 18.49 pg/mL	Atezo + Bev	165	Prospectively	PMID: 36866388
		> 4.77 pg/mL	Atezo + Bev	34	Prospectively	PMID: 35205631
		> 7.4 pg/mL	Atezo + Bev	94	Retrospectively	PMID: 38943340
		> 9.2 pg/mL	Atezo + Bev	96	Retrospectively	PMID: 39652104
	CXCL9	< 419.1 pg/mL	Atezo + Bev	134	Retrospectively	PMID: 38963640
		Low	anti-PD-1 antibody	211	Not mentioned	PMID: 38737384
		< 333 pg/mL	Atezo + Bev	68	Retrospectively	PMID: 37325489
	LAG-3	> 3736.3 pg/mL	Atezo + Bev	134	Retrospectively	PMID: 38963640
	Osteopontin	≥ 61.375 ng/mL	Atezo + Bev	70	Retrospectively	PMID: 36991155
	Plasma growth hormone	> 3.7 μg/L (female) > 0.9 μg/L (male)	Durva + Treme	16	Prospectively	PMID: 38463542
		> 3.7 μg/L (female) > 0.9 μg/L (male)	Atezo + Bev	37	Prospectively	PMID: 36473155
	γδ + Vδ2 + PD1 + T cells and γδ + Vδ2 + Tim3 + T cells	High	camrelizumab + lenvatinib	40	Retrospectively	PMID: 39052085
	CD8+ and CD8+ PD-L1+ peripheral lymphocytes	Low	Atezo + Bev	37	Prospectively	PMID: 37990855
	PD1+ peripheral neutrophils	High	Atezo + Bev	34	Prospectively	PMID: 36980547
	CD8+ central memory T (TCM) cells	Low	Atezo + Bev	83	Retrospectively	PMID: 38611007

*AFP* α-fetoprotein, *DCP* des-gamma-carboxy prothrombin, *CRP* C-reactive protein, *IL-6* Interleukin-6, *CXCL9* chemokine (C-X-C motif) ligand 9, *LAG-3* lymphocyte activation gene 3, *ALBI* albumin-bilirubin, *Atezo + Bev* atezolizumab plus bevacizumab, *Durva + Treme* durvalumab plus tremelimumab

### ALBI score/grade

The ALBI score/grade can be calculated using parameters routinely measured in clinical practice. The ALBI grade has been associated with OS and response rates in cohorts treated with ipilimumab combined with nivolumab or pembrolizumab [11] and in a pooled cohort of various ICI therapies (*n* = 341, 85% Anti-PD[L]-1 monotherapy) [12].

In Atezo + Bev therapy, studies such as Ohama et al.'s investigation (*n* = 719) [13] and Ochi et al.'s analysis (*n* = 242) [14] reported that the ALBI grade contributes to clinical outcomes, including OS and PFS.

On the other hand, in Durva + Treme therapy, the studies by Hiraoka et al. (183 cases) [15] and Fujiwara et al. (22 cases) found no association between mALBI grade/score and PFS [16]. Similarly, Fujii et al.'s study (*n* = 21) [17] showed no difference in response rates based on ALBI grade.

It has been suggested that the observed differences may stem from the fact that ALBI grade worsens during Atezo + Bev therapy [18], whereas it remains stable during Durva + Treme therapy [19–21].

### Alpha-fetoprotein (AFP)

AFP is the tumor marker with the highest level of evidence for HCC and has been extensively studied in relation to the efficacy of ICIs. In a meta-analysis by Zhang et al., the relationship between pre-treatment AFP levels and OS was analyzed across 28 studies involving 3402 cases, yielding a pooled hazard ratio (HR) of 1.689 (95% confidence interval [CI] 1.508–1.893, *p* < 0.001). Additionally, the relationship between pre-treatment AFP levels and PFS was analyzed in 23 studies involving 2740 cases, showing that patients with high AFP levels had shorter PFS (HR: 1.380, 95% CI 1.186–1.607, *p* < 0.001) compared to those with low AFP levels. However, no significant correlation was found between baseline AFP levels and the objective response rate (ORR) in ICI-treated HCC patients (8 studies, 1043 individuals; odds ratio (OR): 0.963, 95% CI 0.710–1.306, *p* = 0.933; *I*^2^ = 10.0%, *p* = 0.353) [22].

In general, studies on Atezo + Bev combination therapy suggest that cases with high AFP levels have a poorer prognosis [13, 23, 24]. On the other hand, Saeki et al. reported that in a study of 110 cases treated with Durva + Treme, the ORR was 8.5% for patients with AFP < 400 ng/mL and 28.2% for those with AFP ≥ 400 ng/mL (*p* = 0.011), indicating better responses in patients with higher AFP levels [25].

In cases with high AFP levels, regardless of treatment regimen, tumor growth tends to be more rapid, and prognosis is generally shorter. Therefore, it is crucial to evaluate the therapeutic benefit of ICIs compared to other treatment options. Data from clinical trials summarizing the therapeutic benefits compared to other standard treatments are presented in Table 2.

**Table 2:** Clinical benefit of ICI according to AFP status

Trial	Phase	Treatment	Target	Notes	Endpoint	AFP, ng/mL	HR(95%CI)	Which emphasizes the benefits of ICI?	PMID
IMbrave150	Phase 3	Atezolizumab + bevacizumab vs sorafenib	PD-L1	First line	PFS	< 400	0.52 (0.34–0.81)	Low AFP	PMID: 32402160
					PFS	> 400	0.68 (0.43–1.08)		
					OS	< 400	0.49 (0.36–0.66)	Low AFP	
					OS	> 400	0.79 (0.54–1.16)		
HIMALAYA	Phase 3	STRIDE vs sorafenib	PD-L1 + CTLA4	First line	OS	< 400	0.82 (0.53–1.05)	High AFP	PMID: 38319892
					OS	> 400	0.64 (0.45–0.91)		
		Durvalumab vs sorafenib	PD-L1		OS	< 400	0.78 (0.61–1.01)	High AFP	
					OS	> 400	0.73 (0.53–1.03)		
RATIONALE-301	Phase 3	Tislezumab vs sorafenib	PD-1	First line	OS	< 400	0.81 (0.64–1.02)	Low AFP	PMID: 37796513
					OS	> 400	0.86 (0.65–1.13)		
LEAP-002	Phase 3	Lenvatinib + pembrolizumab vs lenvatinib	PD-1	First line	OS	< 400	0.95 (0.77–1.17)	High AFP	PMID: 38039993
					OS	> 400	0.67 (0.50–0.90)		
CheckMate 459	Phase 3	Nivolumab vs sorafenib	PD-1	First line	OS	< 400	0.98 (0.78–1.24)	High AFP	PMID: 34914889
					OS	> 400	0.67 (0.51–0.88)		
ORIENT-32	Phase 2–3	Sintilimab + a bevacizumab biosimilar (IBI305) vs sorafenib	PD-1	First line, HBV related	PFS	< 400	0·43 (0·32–0·57)	Low AFP	PMID: 34143971
					PFS	> 400	0·75 (0·55–1·03)		
					OS	< 400	0.54 (0.35–0.83)	Low AFP	
					OS	> 400	0.59 (0.41–0.85)		
KEYNOTE-240	Phase 3	Pembrolizumab vs placebo	PD-1	Sorafenib-treated advanced HCC	PFS	< 200	0.63 (0.46–0.86)	Low AFP	PMID: 37901200
					PFS	> 200	0.83 (0.59–1.16)		
					OS	< 200	0.66 (0.49–0.90)	Low AFP	
					OS	> 200	0.88 (0.63–1.23)		

In the IMbrave150 trial, patients with AFP < 400 had lower HRs for PFS and OS with Atezo + Bev compared to sorafenib, indicating stronger therapeutic benefits for patients with lower AFP levels [5].

Conversely, in the HIMALAYA trial, patients with AFP < 400 experienced a smaller OS benefit from STRIDE compared to sorafenib than did those with AFP ≥ 400 [6].

Similarly, in the LEAP-002 [26] and CheckMate 459 [27] trials, patients with lower AFP levels appeared to derive less benefit. These findings highlight that the utility of baseline AFP as a biomarker remains a topic of ongoing debate.

### C-reactive protein and alpha fetoprotein in immunotherapy (CRAFITY) score

The CRAFITY score was initially reported by Scheiner et al.[28], who analyzed 292 HCC patients receiving PD(L)1-based immunotherapy at any line of systemic therapy. They found that C-reactive protein (CRP) ≥ 1 mg/dL and AFP ≥ 100 ng/mL were independently and significantly associated with OS. Based on these results, patients were categorized into three groups: 0 (AFP < 100 ng/mL and CRP < 1 mg/dL), 1 (either AFP ≥ 100 ng/mL or CRP ≥ 1 mg/dL), or 2 (AFP ≥ 100 ng/mL and CRP ≥ 1 mg/dL).

The utility of the CRAFITY score has been validated in multiple cohorts. Ueno et al. analyzed 302 patients treated with Atezo + Bev (regardless of frontline or later-line therapy) and found that when treatment resistance (defined as the best overall response (BOR) of progressive disease (PD) or stable disease with a PFS of < 180 days) and treatment benefit (defined as either a complete or partial response or stable disease lasting ≥ 180 days) were assessed, the resistance rates in patients with CRAFITY scores of 0, 1, and 2 were 24.6%, 44.6%, and 57.9%, respectively (*p* < 0.001) [29]. Furthermore, this scoring system effectively stratified OS and PFS.

Hatanaka et al. also demonstrated that the CRAFITY score effectively stratified PFS and OS in a study of 297 patients treated with Atezo + Bev (regardless of frontline or later-line therapy) [23]. The median PFS in the CRAFITY score 0, 1, and 2 groups was 11.8 months (95% CI 6.4–not applicable [NA]), 6.5 months (95% CI 4.6–8.0), and 3.2 months (95% CI 1.9–5.0), respectively (*p* < 0.001). The median OS in patients with CRAFITY scores of 0, 1, and 2 was not reached, 14.3 months (95% CI 10.5–NA), and 11.6 months (95% CI 4.9–NA), respectively. They also found that adverse events increased in the order of CRAFITY scores 0, 1, and 2.

### The neutrophil-to-lymphocyte ratio (NLR) and platelet-to-lymphocyte ratio (PLR)

NLR and PLR have garnered attention as promising prognostic markers in various malignancies, including HCC. These ratios are calculated by dividing the absolute neutrophil count or absolute platelet count by the absolute lymphocyte count, and they can be easily derived from routine complete blood count (CBC) results. Neutrophils and platelets may significantly contribute to tumor growth and progression by producing and releasing inflammatory cytokines and growth factors, such as VEGF. In contrast, lymphocytes play a crucial role in the development of anti-tumor immunity through adaptive immune responses. Therefore, these ratios are considered to reflect the influence of systemic inflammatory responses on cancer progression.

#### NLR

High NLR, in particular, indicates a relatively tumor-promoting environment with suppressed tumor immunity, which is thought to limit the benefits of ICI therapy. A meta-analysis by Zhang et al. on ICIs in HCC reported that high NLR was associated with shorter OS (15 studies, 2172 cases, HR: 1.951, 95% CI 1.624–2.343, *p* < 0.001), shorter PFS (13 studies, 1753 cases, HR: 1.632, 95% CI 1.429–1.865, *p* < 0.001), lower ORR (odds ratio (OR): 0.484, 95% CI 0.335–0.698, *p* < 0.001), and lower DCR (OR: 0.494, 95% CI 0.264–0.922, *p* = 0.027) (7 studies, 1080 cases)[22].

To summarize the previous reports without distinguishing between therapeutic agents, high NLR has been reported to correlate with shorter PFS [30–34], shorter OS [30–34], shorter time to progression (TTP) [34], and reduced ORR and DCR [31].

In a study stratified by therapeutic agent, both Atezo + Bev [14, 30, 32, 34] and Durva + Treme [35, 36] were reported to show a decreased treatment benefit due to high NLR.

Moreover, some studies have suggested that early dynamic changes in NLR [37] or NLR at the start of the second treatment cycle [38] are more critical than baseline NLR.

#### PLR

In a retrospective study by Muhammed et al. involving 362 patients treated with ICIs, it was reported that patients with a PLR of 300 or higher had shorter OS (6.4 months vs. 16.5 months, *p* < 0.0001) and PFS (1.8 months vs. 3.7 months, *p* = 0.0006) [33]. PLR remained an independent prognostic factor for both OS and PFS (HR: 1.60, 95% CI 1.16–2.40, *p* = 0.020; HR: 1.99, 95% CI 1.11–3.49, *p* = 0.021).

### Cutoff values

The cutoff values for NLR vary across studies. Examples of proposed cutoffs include 2.56 (Japan) [14], 2.77 (China) [31], 3 (a multinational study involving Italy, Germany, Portugal, Japan, and Korea; 885 cases, 89% from Asian centers, Atezo + Bev) [34], 3.2 (Germany) [30], 5 (14 centers across the U.S., Europe, and Asia; 296 cases, Atezo + Bev) [32], 5 (13 cancer centers across Europe [28%], North America [57%], and Asia [15%]) [33].

### Racial differences in NLR

It is believed that normal NLR values differ by race. Since the counts of leukocytes and neutrophils vary significantly among racial groups, NLR also differs among Caucasians, Hispanics, African Americans, and Asians.

In a study of patients with non-ST-segment elevation myocardial infarction, the NLR of African American patients was significantly lower compared to that of Caucasian patients [39]. Similarly, another study showed that non-Hispanic Black and Hispanic participants had significantly lower mean NLR values compared to non-Hispanic White participants (1.76, 95% CI 1.71–1.81, and 2.08, 95% CI 2.04–2.12, respectively; *p* < 0.0001) [40].

### Other indices and scores

#### Alpha-FAtE

Scored based on the variables AFP < 400 ng/mL, alkaline phosphatase (ALP) < 125 IU/L, and eosinophil count ≥ 70/μL, alpha-FAtE is useful for stratifying OS (HR 0.44, *p* = 0.0009) and TTP (HR 0.34, *p* < 0.0001) in Atezo + Bev treatment[41].

#### IMABALI-De score

Calculated from AFP ≥ 100 ng/mL (HR 1.4; 1 point), modified albumin–bilirubin mALBI 2a (HR 1.7; 1 point), mALBI 2b/3 (HR 2.8; 2 points), and des-gamma-carboxy prothrombin (DCP) ≥ 100 mAU/mL (HR 1.6; 1 point), the IMABALI-De score is useful for stratifying OS (*p* < 0.001; AIC 2788.67, c-index 0.699) and PFS (*p* < 0.001; AIC 5203.32, c-index 0.623) in Atezo + Bev treatment [13].

#### Circulating immune index (CII)

The CII is calculated by dividing the white blood cell count (× 10⁹/L) by the lymphocyte ratio (%). CII may be an independent prognostic factor for OS in combination therapy with ICI (nivolumab, camrelizumab, sintilimab, pembrolizumab, toripalimab, atezolizumab, or tislelizumab) and lenvatinib (training cohort HR [95% CI] 2.24 [1.17–4.31], *p* = 0.015, validation cohort HR [95% CI] 3.27 [1.33–8.07], *p* = 0.010) [42].

#### Modified albumin–bilirubin grade and α-fetoprotein (mALF) score

This score is calculated based on the mALBI grade and α-fetoprotein ≥ 100 ng/mL, stratifying PFS and OS [24].

### Peripheral blood immune cell subpopulation

#### γδ + Vδ2 + PD1 + T Cells/γδ + Vδ2 + Tim3 + T cells

Zhang and colleagues analyzed lymphocyte subsets using flow cytometry in 40 HCC patients treated with camrelizumab and lenvatinib [43]. They reported that patients who did not achieve disease control had higher proportions of γδ + Vδ2 + PD1 + T cells and γδ + Vδ2 + Tim3 + T cells, which are subsets of cytotoxic γδ T cells, compared to those who achieved disease control.

#### CD8+ PD-L1+ peripheral lymphocytes

In a prospective study by Gramantieri and colleagues, 13 HCC patients treated with atezolizumab plus bevacizumab demonstrated objective responses, 17 achieved stable disease, and 7 were classified as non-responders. The proportions of CD8+ and CD8+ PD-L1+ peripheral lymphocytes before treatment were significantly lower in the responder group compared to the non-responder group (*t*-test, *p* = 0.012 and 0.004, respectively) [44].

#### PD1+ peripheral neutrophils

The same research group mentioned above conducted a prospective study on an Italian cohort of 34 patients treated with atezolizumab plus bevacizumab. They measured the proportion of peripheral neutrophils and their PD1 and PD-L1 expression levels before treatment. The proportion of PD1+ peripheral neutrophils before treatment was significantly lower in the responder group compared to the non-responder group (PD1+ neutrophils in responders vs. non-responders: 9.9 ± 9.1% vs. 29.2 ± 17.6%, Student’s *t*-test, *p* < 0.01). Additionally, patients with lower PD1+ neutrophil proportions had a longer TTP (log-rank test, *p* < 0.0001) [45].

#### CD8+ central memory T (TCM) cells and an increase in CD8+ effector memory T (TEM) cell

We have also reported that a higher baseline proportion of CD8 + TCM cells and an increase in the proportion of CD8+ TEM cells after treatment initiation are associated with treatment benefits from Atezo + Bev[46].

#### Serum/plasma proteins interleukin-6 (IL-6)

There have been numerous studies on blood cytokines, with IL-6 being the most frequently reported. Elevated IL-6 levels have been shown to attenuate the therapeutic benefits of Atezo + Bev therapy, particularly in multiple reports from Asia [47–51]. Our research identified a positive correlation between blood IL-6 concentrations and IL-6 expression in cancer cells within HCC tissues [51]. This result suggests that elevated serum IL-6 in these cases originates from cancer cells. Additionally, cases with high blood IL-6 levels exhibited a high TAM/CD8-positive cell ratio, reflecting an immunosuppressive microenvironment [51].

#### Chemokine (C-X-C motif) ligand 9 (CXCL9)

Several studies have suggested that low serum CXCL9 levels are associated with shorter PFS during Atezo + Bev therapy [52–54]. Interestingly, one report noted that while low serum CXCL9 reduces the therapeutic benefits of Atezo + Bev, it correlates with a higher ORR for lenvatinib. Their analysis, utilizing the TCGA database and in vitro studies, demonstrated a significant negative correlation between FGFR4 and CXCL9 expression levels (*R*^2^ = 0.8041, *p* = 0.0155). Furthermore, knocking down FGFR4 in Huh7 cells increased CXCL9 expression, indicating that FGFR4 signaling suppresses CXCL9 gene expression. Considering that lenvatinib is more effective in cases with high FGFR4 expression [55], it is reasonable that cases with low CXCL9 levels respond better to lenvatinib.

#### Lymphocyte activation gene 3 (LAG-3)

High levels of the immune checkpoint molecule LAG-3 in the blood are potentially associated with lower response rates, shorter PFS, and worse OS during Atezo-Bev therapy [52].

#### Osteopontin (OPN or SPP1)

OPN is a secreted, non-collagenous, sialic acid-rich, chemokine-like extracellular matrix (ECM) protein. OPN is a glycosylated phosphoprotein that functions as a cytokine. In a study involving 70 cases of uHCC treated with Atezo + Bev, it was confirmed that the DCR was lower in the high OPN group compared to the low OPN group [56]. Multivariate analysis revealed that high pretreatment OPN levels and high AFP levels were independent predictors of PD. A sub-analysis focusing on patients with Child–Pugh class A showed that PFS was shorter in the high OPN group than in the low OPN group.

#### Growth hormone (GH)

Kaseb and colleagues have studied plasma growth hormone levels in relation to ICI therapies [57, 58]. They reported that levels > 3.7 μg/L in females and > 0.9 μg/L in males reduce the therapeutic benefits of Durva + Treme [58] and Atezo + Bev [57].

## Liquid biopsy

### Circulating tumor DNA

Circulating tumor DNA (ctDNA) is the fraction of the free DNA in the circulation that is derived from primary or metastatic tumors. It is emerging as a potential non-invasive biomarker and is beginning to be used in clinical practice to detect mutations in several major cancer types and to monitor disease progression [59].

In HCC, ctDNA has also been shown to be beneficial for detecting genomic changes in patients at advanced stages [60].

The advantages include non-invasiveness and the ability to overcome sampling bias caused by intratumoral and intertumoral heterogeneity. The analysis of ctDNA is already well-established, and ctDNA panel testing has already been applied in clinical settings.

Therefore, if specific mutations and their association with the efficacy of combination immunotherapies are clarified, ctDNA could provide a non-invasive way to predict treatment effectiveness.

### Mutation profiling in HCC and immunotherapy response

In terms of tissue-based studies, *CTNNB1*-mutated HCC has been reported to induce an immune-cold tumor microenvironment (TME) with a lack of immune cell infiltration, promoting resistance to ICI monotherapy [61]. WNT/β-catenin signaling has been associated with lower DCR (0% vs. 53%) and shorter median PFS (2.0 vs. 7.4 months) in ICI therapy, where 81% of patients received anti-PD-(L)1 monotherapy [62].

On the other hand, although it is speculated that bevacizumab may improve the immunosuppressive tumor microenvironment induced by CTNNB1 mutations, a report found no association between CTNNB1 mutations and objective response rate (ORR), DCR, or PFS in the context of Atezo + Bev treatment [63].

Additionally, tumor mutation burden (TMB), i.e., the number of somatic mutations per megabase of the tumor genome, is generally considered to reflect a higher abundance of neoantigens derived from the tumor and enhanced immunogenicity. TMB-high has recently attracted attention as a biomarker to predict the effectiveness of immune checkpoint inhibitors in cancers like lung, bladder, and head and neck cancers [64].

However, in HCC, inconsistent results have been reported. Secondary analysis of patients from the GO30140 and IMbrave150 trials (76 and 130 patients, respectively, all with advanced HCC and treated with atezolizumab and bevacizumab) evaluated the relationship between baseline TMB and clinical outcomes [10]. In the GO30140 trial, ORR was observed in the highest TMB tertile, but there was no significant difference in PFS across tertiles. In the IMbrave150 trial, no correlation was found between TMB tertiles and objective response, PFS, or OS. Similarly, a retrospective study of 99 patients with advanced HCC treated with nivolumab or pembrolizumab found no correlation between TMB and either PFS or ORR [65].

Regarding research using ctDNA, a retrospective study analyzed ctDNA from 44 HCC patients treated with nivolumab using Guardian Health's panel test. The study found that mutations associated with *PIK3CA*, *BRCA1*, and *CCND1* amplifications were linked to shorter OS. Additionally, mutations in *KIT* and *PIK3CA* were associated with a shorter PFS, while mutations in *CTNNB1* were associated with a longer PFS [66].

Furthermore, a multi-center cohort study of 85 HCC patients treated with atezolizumab and bevacizumab found that 26 patients with *TERT* gene mutations had significantly shorter OS compared to those without *TERT* mutations. Treatment response and PFS were similar in both groups, suggesting that *TERT* mutations may be more closely related to advanced underlying liver disease rather than response to combination immunotherapy [67]. Additionally, the ctDNA profiling in this study did not show any association between specific *CTNNB1* mutations and treatment response or PFS, which aligns with the previous report [63].

### Circulating tumor cells

Circulating tumor cells (CTCs), i.e., intact tumor cells that are present in the circulation, provide various types of information, including abnormal protein expression, genomic mutations, and messenger RNA in solid tumors. While there are few reports on CTCs as biomarkers for immunotherapy in HCC, a study involving 124 cases receiving immune therapy combined with targeted therapy found that cases with PD-L1 (+) CTCs had extended PFS (HR = 6.359) and OS (HR = 6.67) [68].

### Cell-free RNA

Several studies have also shown the prognostic value of circulating miRNAs in HCC. However, there are few reports on circulating RNA as biomarkers for immunotherapy in HCC. One study found that high levels of Lnc-CCNH-8 in plasma exosomes in HCC patients were associated with a favorable treatment response to ICIs. Mechanistically, up-regulated Lnc-CCNH-8 sponges miR-217 to regulate PD-L1 expression. Additionally, Lnc-CCNH-8 stabilizes PD-L1 through the miR-3173/PKP3 axis. In other words, Lnc-CCNH-8 upregulates PD-L1 in a miR-217/miR-3173-dependent manner, evading CD8+ T cell-mediated killing. By overcoming this immune evasion mechanism with ICIs, a therapeutic response can be expected [69].

## Gut microbiome and metabolome

Perturbations of gut microbiota and impairment of the integrity of the mucosal barrier lead to dysbiosis, which is associated with an increased risk of immune-related diseases and cancer [70]. Furthermore, local induction effects of acquired immunity and immune-regulatory cytokines by gut microbiota have been reported to enhance adaptive immune responses, influencing antitumor effects [71]. Representative drugs that alter the gut microbiota include proton pump inhibitors (PPIs) and antibiotics.

In the field of HCC, several reports have linked antibiotic use in patients with poor prognosis after ICI treatment. These include shortened OS with anti-PD1 blockade [72], shortened OS with ICIs (nivolumab, pembrolizumab, or ipilimumab) [73], decreased DCR (no significant difference in ORR), and shortened PFS and OS with Atezo + Bev treatment [74]. Furthermore, while no differences were observed in cohorts including patients treated with various ICI monotherapies or combination immunotherapies, a report showed that, specifically in cases of viral hepatitis, the antibiotic-treated group experienced shortened OS and PFS [75].

On the other hand, in the antibiotic-treated group, worse outcomes have been observed not only with ICIs but also with tyrosine kinase inhibitors and placebos, suggesting caution in interpreting the results [76]. However, there is also a report suggesting that antibiotics administered within 30 days before or after ICI initiation may be associated with improved benefit from immunotherapy [77].

Regarding PPI administration, some reports indicate no difference in prognosis compared to the non-administration group [78–80]. However, a subgroup analysis of antiviral therapy in patients with HBV-DNA levels above 200 IU/mL showed an increased risk of death in the PPI group [79].

Recent studies have highlighted the critical role of the gut microbiome in modulating therapeutic responses to ICIs in several solid tumors, including HCC.

Zhu et al. retrospectively analyzed the gut microbiome, mycobiome (fungal community), and metabolome (metabolic products) of 80 HCC patients treated with ICIs (PD-1/PD-L1 inhibitors, with or without targeted therapy) [81]. Their findings revealed that the diversity of bacteria and fungi before treatment was higher in the durable clinical benefit group compared to the non-durable clinical benefit group, with differences in diversity beginning to emerge 6–8 weeks after immunotherapy initiation. Furthermore, two bacterial species (*Actinomyces_sp_ICM4* and *Senegalimassilia_anaerobia*) and one metabolite (galanthaminone) were identified as useful prognostic biomarkers [81].

Conversely, Inukai et al. examined cases treated with Atezo + Bev and found no significant differences in alpha or beta diversity between responders and non-responders [82]. However, the relative abundance of *Bacteroides stercoris* and *Parabacteroides merdae* was higher in responders than in non-responders. Prognostic analysis based on the presence of these bacteria revealed that patients lacking both species had significantly poorer outcomes [82].

In a prospective study by Lee et al., fecal microbiota and metabolites were analyzed. *Prevotella 9* was enriched in PD patients, while *Lachnoclostridium*, *Lachnospiraceae*, and *Veillonella* were predominant in OR patients. Among metabolites, ursodeoxycholic acid and ursocholic acid were significantly enriched in the feces of OR patients and strongly correlated with the abundance of *Lachnoclostridium*. The coexistence of increased *Lachnoclostridium* and decreased *Prevotella 9* significantly predicted better OS [83].

These findings suggest that pre-treatment gut microbiota and metabolite profiles may serve as potential biomarkers for ICI therapy. However, no common bacterial taxa or metabolites validated across multiple studies have been identified. Additionally, differences in gut microbiota may be influenced by factors such as age and clinical background, including liver functional reserve, necessitating cautious interpretation of results.

Studies involving the association of gut microbiome or metabolites with immunotherapy are summarized in Table 3.

**Table 3:** Gut microbiome/metabolome in immunotherapy

Gut microbiome/metabolome	Categories	Therapeutic outcome	Treatment	*n*	Study design	PMID
*Actinomyces_sp_ICM47*	Bacterial species	Unfavorable	PD-1/PD-L1 inhibitors	80	Prospectively	PMID: 38844407
*Senegalimassilia_anaerobia*	Bacterial species	Unfavorable				
galanthaminone	Metabolite	Favorable				
The diversity of bacteria and fungi		Favorable				
*Bacteroides stercoris*	Bacterial species	Favorable	Atezo + Bev	37	Retrospectively	PMID: 38730627
*Parabacteroides merdae*	Bacterial species	Favorable				
*Prevotella 9*	Bacterial species	Unfavorable	ICI (PD-(L)1 monotherapy: *n* = 24, combination with TKI: *n* = 17)	41	Prospectively	PMID: 35738801
*Lachnoclostridium*	Bacterial species	Favorable				
*Lachnospiraceae*	Bacterial species	Favorable				
*Veillonella*	Bacterial species	Favorable				

## Imaging

In recent years, there has been increasing interest in using medical imaging techniques such as magnetic resonance imaging (MRI), computed tomography (CT), and PET/CT to indirectly or directly predict the tumor microenvironment and subclasses of hepatocellular carcinoma, as well as the efficacy of immunotherapy. The importance of imaging examinations as biomarkers is expected to continue to grow. Candidates for imaging biomarkers are summarized in Table 4.

**Table 4:** Imaging biomarkers

Modality	Findings	Therapeutic outcome	Treatment	*n*	Study design	PMID/DOI
EOB-MRI	High RER	Unfavorable	Anti-PD-1/PD-L1 monotherapy	18 (68 lesions)	Retrospectively	PMID: 34950184
	High RER	Unfavorable	Atezo + Bev	35	Retrospectively	PMID: 35159095
	Heterogeneous signal intensity					
	rim aphe, peritumoral enhancement in arterial phase, or intermediate intensity on HBP	Favorable	Atezo + Bev	27 (60 lesions)	Retrospectively	https://doi.org/10.1159/000542099
Chemical shift MR imaging	Steatohepatic HCC	Favorable	Atezo + Bev	30	Retrospectively	PMID: 35567547
FDG-PET CT	High SUVmax	Favorable	Durva + Treme	21	Retrospectively	PMID: 39527933
Contrast-enhanced CT	Non simple nodular type	Favorable	Atezo + Bev	74 patients (95 lesions)	Retrospectively	PMID: 38353524
	Tumor shape irregularity	Unfavorable	Atezo + Bev	395	Retrospectively	PMID: 39714631
CEUS	Low rising time ratio	Favorable	ICI + anti-angiogenic therapy	66	Prospectively	PMID: 38072718

*EOB-MRI* gadoxetic acid-enhanced MRI, *RER* relative enhancement, *APHE* arterial phase hyperenhancement, *HBP* hepatobiliary phase, *FDG-PET* fluorodeoxyglucose positron emission tomography, *SUVmax* maximum standardized uptake value, *CEUS* contrast-enhanced ultrasound

### MRI

It has long been known that high signal intensity in the hepatobiliary phase of gadolinium-ethoxybenzyl-diethylenetriamine (Gd-EOB-DTPA)-enhanced MRI reflects Wnt/β-catenin activation/mutation. Tumors with Wnt/β-catenin activation/mutation exhibit increased expression of organic anion transporting polypeptide 1B3 (OATP1B3), a transporter that absorbs EOB, resulting in high signal intensity in the hepatobiliary phase (HBP). Since Wnt/β-catenin activation/mutation is associated with an immune-cold tumor microenvironment, various studies have explored the relationship between high signal intensity in the HBP and the efficacy of ICIs [84].

For instance, Aoki and colleagues analyzed 68 intrahepatic HCC nodules from 18 patients treated with anti-PD-1/PD-L1 monotherapy. They reported that patients with high-signal HCC nodules in HBP (*n* = 8) had shorter PFS and TTP compared to patients with low-signal HCC nodules (*n* = 10) [85]. Similarly, Sasaki and colleagues analyzed 68 patients (33 in the lenvatinib group and 35 in the Atezo + Bev combination group). In the Atezo + Bev group, PFS was significantly shorter in patients with high-signal nodules compared to those with low-signal nodules [86].

On the other hand, in our analysis of 65 cases treated with Atezo + Bev, high signal intensity in the HBP was not associated with treatment efficacy [87].

While there is consensus that signal intensity in the HBP is associated with β-catenin mutations or pathway activation, whether it can predict the efficacy of ICIs, particularly combination immunotherapy, remains a matter of debate.

Many studies use the relative enhancement ratio (RER) as an index to measure signal intensity. RER is calculated as the ratio of the tumor-to-background liver parenchyma signal intensity (relative intensity ratio: RIR) in the HBP to that in the precontrast phase using the following formula: RER = RIR in HBP/RIR in the precontrast phase.

However, the signal intensity of the background liver parenchyma in the HBP may be influenced by liver function reserve, which poses a challenge.

Some studies focus not only on signal intensity but also on its uniformity. It has been suggested that heterogeneous signal intensity types may respond less effectively to Atezo + Bev compared to homogeneous signal intensity types [86].

Additionally, certain features in the hepatobiliary phase of EOB-MRI may predict treatment outcomes. The absence of an enhancing capsule (AEC) is associated with CD8-positive cell density in the tumor, while irregular tumor margins (ITM) in the hepatobiliary phase are linked to PD-L1 expression. Peritumoral low signal intensity (PLSI) in the hepatobiliary phase is associated with both PD-L1 expression and CD8 density. The combination of PLSI and AEC or PLSI and ITM may allow for the pre-treatment prediction of anti-PD-1 therapy efficacy [88].

Steatotic HCC, which accounts for 23% of non-viral HCC cases, is characterized by T-cell exhaustion, infiltration of M2 macrophages and cancer-associated fibroblasts, high PD-L1 expression, and TGF-β signaling activation. This immune-enriched but exhausted tumor immune microenvironment is associated with significantly longer PFS in patients treated with combined immunotherapy using anti-PD-L1 and anti-VEGF antibodies, as confirmed by chemical-shift MR imaging [89].

Ueshima et al. analyzed preoperative EOB-MRI in patients with HCC who underwent surgical resection and identified three imaging findings associated with the immune-excluded type of HCC [90]:

1. Rim arterial phase hyperenhancement (rim APHE) (OR 17.3, *p* = 0.009).
2. Peritumoral enhancement in the arterial phase (OR 8.6, *p* < 0.004).
3. Intermediate intensity in the HBP (three-tiered visual assessment scale) (OR 28.2, *p* = 0.002).

Spatial transcriptomic analysis revealed that in immune-excluded HCC, there was a correlation between cytotoxic T lymphocytes (CTLs), VEGF signaling, and cancer-associated fibroblasts (CAFs) at the invasive margin (IM) of the tumor.

Furthermore, an analysis of treatment response in 27 patients (60 lesions) who received Atezo + Bev showed a significantly prolonged time to progression (TTP) in cases exhibiting any of the three imaging findings above.

These results suggest that blocking the VEGF pathway may facilitate CTL infiltration into the tumor, thereby enhancing the efficacy of ICIs against the immune-excluded phenotype.

In conclusion, enhancing predictive accuracy by incorporating multiple factors is considered an effective approach.

### Positron emission tomography (PET)/CT

One promising imaging modality for predicting the immune environment of HCC, alongside EOB-MRI, is PET/CT. In HCC, FDG uptake has been reported to positively correlate with CD8+ T cell counts and CD68+ macrophage infiltration [91, 92], as well as with PD-L1 expression [92], supporting its reflection of the immune microenvironment. Activated immune cells, such as leukocytes, exhibit increased energy consumption and actively take up fluorodeoxyglucose (FDG), a glucose analog used as a PET tracer [93].

We retrospectively evaluated 21 patients with uHCC who received the STRIDE regimen as first-line therapy and reported that a high tumor-to-liver ratio (TLR) of maximum standardized uptake value (SUVmax) was associated with treatment efficacy [17]. However, further investigation is needed to clarify the relationship between SUVmax and the benefits of ICIs in HCC.

### Ultrasound (US)

Ultrasound is a non-invasive diagnostic tool useful for differential diagnosis. While there are fewer reports on its role in predicting the efficacy and clinical benefits of ICIs compared to MRI or PET/CT, Zhang et al. conducted a prospective study involving 66 HCC patients treated with a combination of ICIs and anti-angiogenic therapy. Pre-treatment contrast-enhanced ultrasound (CEUS) was performed, and multivariate logistic regression analysis identified the rising time (RT) ratio as the only independent factor associated with the ORR (OR = 0.007, 95% CI 0.000–0.307, *p* = 0.010). Survival analysis demonstrated that patients with a lower RT ratio had significantly longer PFS (low RT ratio vs. high RT ratio = not reached vs. 4.4 months, *p* = 0.001) [94].

### Contrast-enhanced CT

The relationship between contrast-enhanced CT imaging and the therapeutic efficacy or clinical benefits of ICIs has been reported by several groups. Ishihara et al. analyzed 95 intrahepatic lesions in 74 patients treated with Atezo + Bev. These lesions were categorized into two groups based on their macroscopic morphology observed in pre-treatment imaging: simple nodular (SN) type and non-SN type. Treatment responses and other related clinical outcomes were evaluated. An assessment of post-treatment tumor sizes revealed that the tumor reduction rate was higher in the non-SN group, which included 56 lesions, compared to the SN group, which included 39 lesions. The ORR was significantly higher in the non-SN group (39.3% vs. 15.4%, *p* = 0.012). Additionally, the median time to tumor progression was longer in the non-SN group (21.0 months vs. 8.1 months, *p* = 0.119) [95].

The poor prognosis feature of tumor shape irregularity was also reported by Zhang et al. [96].

Furthermore, there have been reports on the usefulness of artificial intelligence (AI) and deep learning models based on imaging data in predicting the response to ICIs [97, 98].

### Multiple modalities approach

Zhang et al. introduced the AFP and Tumor Shape Irregularity (ATSI) score, which evaluates the number of baseline conditions—AFP ≥ 400 ng/mL and initial tumor shape irregularity—met by a patient, based on a training set of 177 individuals who had poor prognoses with Atezo + Bev treatment [96]. Using this scoring system, they validated their findings on a set of 119 patients. Kaplan–Meier survival curves based on ATSI scores revealed significant differences in PFS and OS among the three patient groups. The median OS for patients with an ATSI score of 0 points (*n* = 37) was 37.43 months (95% CI 28.10–NA), significantly better than those with 1 point (*n* = 48) at 24.27 months (95% CI 18.23–NA) and those with 2 points (*n* = 34) at 14.03 months (95% CI 12.37–NA) (*p* = 0.028). The median PFS was 13.93 months (95% CI 10.73–NA) for 0 points, 8.30 months (95% CI 6.73–16.33) for 1 point, and 4.90 months (95% CI 3.00–9.03) for 2 points. Additionally, the ORR/DCR was 27.78%/86.11%, 23.91%/78.26%, and 17.65%/55.88% for scores of 0, 1, and 2 points, respectively.

Yang and colleagues proposed the GRAPHS-CRAFITY score, calculated from CRAFITY, gender, and MRI findings (gross growth types [non-infiltrative type or infiltrative type], enhancing tumor capsule [complete/partial/without], and intratumoral fat [yes or no]) [99].

## Conclusions

This review comprehensively examines biomarkers related to treatment outcomes of ICI therapy for HCC, including blood, imaging, gut microbiota, and clinical background. The advantages and disadvantages of each marker are summarized in Table 5.

**Table 5:** Advantages and disadvantages of each class of biomarker

	Advantages	Issues
Conventional tumor markers, e.g., AFP, DCP	Measurable in routine clinical practice	Not a specific marker of the immune microenvironment. Influenced by tumor burden
Scoring systems, e.g., CRAFITY score	Measurable in routine clinical practice	Not a specific marker of the immune microenvironment. Limited reports on anti-PD-(L)1 + anti-CTLA4
(m)ALBI	Measurable in routine clinical practice	It is a marker of liver reserve function. Cases with poor liver reserve have a poor prognosis regardless of the treatment chosen
Neutrophil-to-lymphocyte ratio (NLR)	Measurable in routine clinical practice	There are racial differences. It may be influenced by factors other than the tumor
Cytokines	The measurement method is well established. Some of the cytokines are measurable in routine clinical practice	The condition of the background liver; comorbidities and tumor burden may have an impact
Peripheral blood immunoprofiling	It may reflect the systemic immune system	There are hurdles to applying this in daily clinical practice. There are hardly any findings that have been validated in different institutions
Liquid biopsy, especially ctDNA	The measurement techniques are well-established, and there are panels that are already in clinical use	The association between mutations and the efficacy of ICIs remains controversial
Gut microbiome	It is the least invasive	There are racial differences. In the field of HCC, there are few results that have been validated across institutions
Imaging	Measurable in routine clinical practice	The signal intensity in the hepatocyte phase of EOB-MRI is influenced by liver function. Quantitative values such as signal intensity vary depending on the equipment and imaging conditions, making direct application difficult. In cases with multiple lesions, there is the issue of which lesion to use for evaluation

*AFP* alpha-fetoprotein, *DCP* Des-gamma-carboxy prothrombin, *CRAFITY* C-reactive protein and alpha fetoprotein in immunotherapy, *(m)ALBI* (modified) albumin-bilirubin, *NLR* neutrophil-to-lymphocyte ratio, *EOB-MRI* gadoxetic acid-enhanced MRI

Research on predicting the therapeutic effects of ICIs has made steady progress, but a definitive, established biomarker has yet to be identified. At this point, combining multiple modalities may be the key to achieving more accurate predictions. For instance, the approach proposed by Zhang et al. [96] and Yang et al. [99], noted above, could serve as a good example.

However, the development of biomarkers that not only predict prognosis but also assist in treatment strategy selection and optimization is essential. In future studies, specific biomarkers are expected to serve as indicators of which treatment is most effective, becoming an important tool in advancing personalized medicine. In particular, the refinement of biomarkers to clarify treatment options and provide the most suitable therapy for patients is urgently needed. This will pave the way for maximizing the efficacy of immunotherapy and improving patient survival rates.

## Abbreviations

ICIs: Immune checkpoint inhibitors
uHCC: Unresectable hepatocellular carcinoma
ASCO: The American Society of Clinical Oncology
PD-L1: Programmed death ligand 1
VEGF: Vascular endothelial growth factor
Atezo: Atezolizumab
Bev: Bevacizumab
Durva: Durvalumab
Treme: Tremelimumab
CTLA4: Cytotoxic T lymphocyte-associated antigen 4
ECOG: Eastern Cooperative Oncology Group
ESMO: European Society for Medical Oncology
BCLC: Barcelona Clinic Liver Cancer
EASL: European Association for the Study of the Liver
AASLD: American Association for the Study of Liver Diseases
PS: Performance status
TACE: Transarterial chemoembolization
RT: Ablation, and radiation therapy
OS: Overall survival
PFS: Progression-free survival
ALBI: Albumin–bilirubin
AFP: Alpha-fetoprotein
HR: Hazard ratio
DCP: Des-gamma-carboxy prothrombin
CI: Confidence interval
ORR: Objective response rate
OR: Odds ratio
CRAFITY: C-reactive protein and alpha fetoprotein in immunotherapy
BOR: Best overall response
PD: Progressive disease
NA: Not applicable
NLR: Neutrophil-to-lymphocyte ratio
PLR: Platelet-to-lymphocyte ratio
CBC: Complete blood count
TTP: Time to progression
ALP: Alkaline phosphatase
CII: Circulating immune index
mALF: Modified albumin–bilirubin grade and α-fetoprotein
TCM: Central memory T
TEM: Effector memory T
IL-6: Interleukin-6
CXCL9: Chemokine (C-X-C motif) ligand 9
LAG-3: Lymphocyte activation gene 3
OPN: Osteopontin
GH: Growth hormone
ctDNA: Circulating tumor DNA
TME: Tumor microenvironment
MRI: Magnetic resonance imaging
CT: Computed tomography
OATP1B3: Organic anion transporting polypeptide 1B3
HBP: Hepatobiliary phase
RER: Relative enhancement
RIR: Relative intensity ratio
AEC: Absence of an enhancing capsule
ITM: Irregular tumor margins
PLSI: Peritumoral low signal intensity
CTLs: Cytotoxic T lymphocytes
CAFs: Cancer-associated fibroblasts
IM: Invasive margin
TTP: Time to progression
CTCs: Circulating tumor cells
PET: Positron emission tomography
FDG: Fluorodeoxyglucose
TLR: Tumor-to-liver ratio
SUVmax: Maximum standardized uptake value
US: Ultrasound
CEUS: Contrast-enhanced ultrasound
RT: Rising time
SN: Simple nodular
AI: Artificial intelligence
ATSI: AFP and tumor shape irregularity
